# Transforming Dental Care, Practice and Education with Additive Manufacturing and 3D Printing: Innovations in Materials, Technologies, and Future Pathways

**DOI:** 10.3390/dj13120555

**Published:** 2025-11-25

**Authors:** Shilthia Monalisa, Mahdieh Alipuor, Debangshu Paul, Md Ataur Rahman, Nazeeba Siddika, Ehsanul Hoque Apu, Rubayet Bin Mostafiz

**Affiliations:** 1 Department of Public Administration, Louisiana State University, Baton Rouge, LA 70820, USA; 2 LaHouse Research and Education Center, Louisiana State University Agricultural Center, Baton Rouge, LA 70820, USA; rmostafiz@agcenter.lsu.edu; 3 College of Dental Medicine, Lincoln Memorial University, Knoxville, TN 37923, USA; mahdieh.alipour@lmunet.edu; 4 Department of Civil and Environmental Engineering, University of Tennessee, Knoxville, TN 37996, USA; dpaul11@vols.utk.edu; 5 Department of Oncology, Karmanos Cancer Institute, Wayne State University, Detroit, MI 48201, USA; ataur1981rahman@hotmail.com; 6 Department of Environmental Health Sciences, School of Public Health, University of Michigan, Ann Arbor, MI 48109, USA; nsiddika@umich.edu; 7 Bitonte College of Dentistry, Northeast Ohio Medical University, Rootstown, OH 44272, USA; ehoqueapu@neomed.edu; 8 Department of Biomedical Sciences, Northeast Ohio Medical University, Rootstown, OH 44272, USA

**Keywords:** additive manufacturing, 3D printing, dental prosthetics, biocompatible materials, digital dentistry, regenerative applications

## Abstract

Additive manufacturing (AM), commonly known as 3D printing, is revolutionizing modern dentistry, introducing high-precision, patient-specific, and digital-driven workflows across prosthodontics, orthodontics, implantology, and maxillofacial surgery. Extensive analysis explores the leading platforms in 3D printing such as stereolithography (SLA), fused deposition modeling (FDM), selective laser sintering (SLS), digital light processing (DLP), and PolyJet which all achieve superior performance across multiple areas including resolution capabilities, material compatibility options, clinical application readiness, and cost-effectiveness. Additionally, an extensive overview of common materials, including biocompatible polymers (PLA, PMMA, PEEK), metals (titanium, cobalt-chromium), and ceramics (zirconia, alumina, glass-ceramics), sheds light on the critical role of material selection for patient safety, durability, and functional performance. The review explores new advancements such as 4D printing with shape-adaptive smart biomaterials as well as artificial intelligence-enabled digital processes and prosthesis design for the transformation of regenerative dentistry and intraoral drug delivery operations into new domains and the automation of clinical planning. Equally groundbreaking are 3D printing applications in pediatric dentistry, surgical simulation, and dental education. However, full-scale adoption of AM technology is not without challenges, including material toxicity, regulatory hurdles for approval, high initial investments, and the need for extensive digital expertise training. Sustainability concerns are also being addressed, with recycled materials and circular economy models gaining traction. In conclusion, this article advocates for a future where dentistry is shaped by interdisciplinary collaboration, intelligent automation, and hyper-personalized biocompatible solutions, with 3D printing firmly established as the backbone of next-generation dental care.

## 1. Introduction

Additive manufacturing (AM), also known as 3D printing, is rapidly revolutionizing dental science by enabling unparalleled precision, personalization, and workflow efficiency in clinical practice. In contrast to traditional subtractive techniques, additive manufacturing fabricates dental items incrementally from digital models, minimizing material waste and production duration while enabling intricate geometries [1]. This technological advancement enhances treatment precision and significantly improves individualized patient care across various dental specialties [2].

The implementation of digital workflows such as intraoral scanning, computer-aided design (CAD), and computer-aided manufacturing (CAM) has significantly expedited the incorporation of 3D printing into dental practice [3]. These advances are transforming the production of crowns, bridges, surgical guides, orthodontic devices, and maxillofacial prostheses by enhancing accuracy, expediting manufacturing, and tailoring them to specific anatomical needs [4,5].

Numerous 3D printing technologies such as stereolithography (SLA), fused deposition modeling (FDM), selective laser sintering (SLS), and digital light processing (DLP) are extensively utilized in dental applications, each presenting distinct advantages in resolution, speed, and material compatibility [6,7]. Advancements in biocompatible materials, including polymers, ceramics, and metal alloys, enhancing the scope and dependability of dental applications, ranging from temporary models to permanent restorations and tissue scaffolds [8,9]. The fundamentals of 3D printing in biomedical engineering underscore its interdisciplinary nature, enabling the creation of custom implants and prosthetics that bridge engineering innovation with clinical application [10].

Furthermore, to date, no recent comprehensive review has been conducted to evaluate the latest publications in this field. Therefore, the present review aims to summarize and discuss recent advances in the application of additive manufacturing in dentistry. The primary objective of this review is to provide a comprehensive examination of additive manufacturing in dental science, focusing on contemporary materials, printing technologies, and clinical applications. Additionally, it examines emerging breakthroughs, such as 4D printing and the integration of artificial intelligence (AI), as well as the prevailing challenges and prospective trajectories that will shape the forthcoming evolution of dental care.

## 2. Additive Manufacturing Techniques in Dentistry

### 2.1. Overview of 3D Printing Technologies

Additive manufacturing (AM) in dentistry encompasses various 3D printing technologies with distinct mechanisms, materials, and applications. The most used techniques include SLA, FDM, SLS, DLP, PolyJet, and others. SLA uses lasers to cure liquid resin layer by layer, producing high-resolution, smooth-surfaced models. It is widely used for surgical guides, crowns, and bridges due to its precision [5,7]. FDM extrudes thermoplastic filaments through a heated nozzle. It is cost-effective and suitable for educational models and preliminary prototypes, although it offers lower resolution than SLA or DLP [11]. SLS uses a laser to convert powdered materials into solid structures, typically nylon or metal. It is valued for its strength and is often used in the production of durable dental prosthetics and frameworks [12]. Like SLA, DLP uses a digital light projector to cure the resin. It is faster than SLA and provides excellent resolution, making it ideal for detailed dental restorations [3,6]. PolyJet technology jets photopolymer layers onto a build platform and cures them with UV light. It enables multi-material and multi-color printing, making it particularly useful for anatomical models and complex prosthetics [13].

### 2.2. Comparative Analysis of Techniques

Comparative studies have shown that the trueness of dental master casts can vary significantly depending on the 3D printing technology used, underscoring the importance of selecting appropriate methods for high-precision applications [14]. Table 1 is showing how each 3D printing method offers unique advantages and limitations in terms of accuracy, resolution, speed, and cost, which influence their suitability for specific dental applications:

The choice of technology depends on the clinical requirements, budget, and desired material properties. For instance, SLA and DLP are preferred for high-precision restorations, while FDM is often used in educational settings due to its affordability and ease of use [6,7].

## 3. Materials Used in Dental 3D Printing

### 3.1. Polymers

Polymers are among the most widely used materials in dental 3D printing due to their versatility, biocompatibility, and ease of processing. Commonly used polymers include polylactic acid (PLA), polymethyl methacrylate (PMMA), polyether ether ketone (PEEK), and resins. PLA is a biodegradable thermoplastic derived from renewable resources and is frequently used in FDM printing for educational models and temporary restorations [8]. PMMA is known for its aesthetic properties and biocompatibility. PMMA fabricates dentures and provisional crowns [5]. PEEK is a high-performance polymer known for its exceptional mechanical strength and chemical resistance, significantly enhancing the quality and durability of dental prostheses and implants. It has been suggested that a CAD-CAM three-piece PEEK FDP has a higher tensile strength compared to a compacted granular- or pellet- shaped PEEK denture [16]. Photopolymer resins are extensively used in SLA and DLP printing for producing surgical guides, crowns, and bridges due to their high resolution and smooth finish [15].

### 3.2. Metals

Metals are essential in dental applications requiring high strength and durability. The most used metals are titanium and cobalt-chrome alloys. Titanium is valued for its biocompatibility and corrosion resistance and is widely used in dental implants and frameworks [17]. Cobalt-chrome alloys offer excellent mechanical properties and are used in removable partial dentures and fixed prosthodontics [8].

### 3.3. Ceramics and Composites

3D printing continues to evolve in restorative dentistry, with applications now extending beyond diagnostic models to include interim and permanent restorations, occlusal splints, and maxillofacial prosthetics, supported by ongoing advancements in ceramic and hybrid materials [18]. Ceramics are favored in restorative dentistry for their aesthetic qualities and wear resistance. Key materials are zirconia, alumina, and glass ceramics. Zirconia is renowned for its exceptional strength and its resemblance to tooth enamel. Zirconia is used in crowns, bridges, and implant abutments [8]. Alumina offers high hardness and is used in ceramic restorations where aesthetics is critical [5]. Glass ceramics materials combine the translucency of glass with the strength of ceramics, making them ideal for anterior restorations [6].

### 3.4. Biocompatibility and Testing Challenges

Many materials used in healthcare 3D printing from polymers to composites are being evaluated for their biocompatibility, mechanical strength, and suitability for specific clinical applications, including dental restorations and implants [19]. Biomaterials used in 3D printing are continually evolving to meet the demands of dental applications, with a focus on enhancing biocompatibility, mechanical strength, and adaptability to address diverse clinical needs [20]. Despite the advancements in materials, ensuring biocompatibility remains a critical challenge. Key concerns are:

- Cytotoxicity: Residual monomers from incomplete polymerization can lead to cytotoxic effects, especially in resins [21].
- Post-Processing Requirements: Proper curing, cleaning, and sterilization are essential to minimize toxicity and ensure mechanical integrity [15].
- Regulatory Compliance: Materials must meet stringent standards for safety and efficacy, which can vary by region and application [22].

Table 2 is a summary table comparing the primary categories of materials used in 3D printing for dental applications, and it provides a quick reference for selecting materials based on clinical needs, balancing performance, aesthetics, and cost.

Ongoing research is focused on developing new materials with enhanced biocompatibility, improved mechanical properties, and increased environmental sustainability. Recent findings suggest that the mechanical performance and surface quality of 3D-printed provisional fixed dental prostheses are significantly influenced by printing orientation, material composition, and post-processing protocols, with horizontal orientations and zirconia-filled resins yielding superior outcomes [23].

## 4. Applications in Dental Practice

### 4.1. Prosthodontics

3D printing has significantly advanced prosthodontics by enabling the fabrication of highly accurate and customized dental restorations [24]. Additive manufacturing has demonstrated significant potential across biomedical fields, particularly in producing customized prosthetics, implants, and scaffolds, capabilities that are increasingly being leveraged in dental applications for both functional and aesthetic outcomes [25]. Common applications are:

- Crowns and Bridges: Printed using biocompatible resins and ceramics for high precision and aesthetics [15].
- Dentures: Rapid prototyping enables a quicker turnaround and a better fit, thereby improving patient comfort and satisfaction [5].

### 4.2. Orthodontics

In orthodontics, some types of 3D printers are commonly used, such as, stereolithography (SLA), FDM, digital light procession (DLP), polyjet photopolymer (PPP), and selective laser sintering (SLS) [26]. Overall, the use of AM technology and 3D printers alongside intraoral scanners eliminates the need for tedious dental impressions and lengthy manual processes, such as gypsum casting, in some orthodontic workflows, resulting in a reproducible, fast, digitalized workflow. The digital fast and robust workflow guides the AM, 3D printing techniques open precision care through more patient-specific designs, enabling doctors to develop new solutions for complicated cases [27]. A large percentage of the research on AM in orthodontics has focused on the accuracy and robustness of 3D-printed orthodontic models. For constructive appliances, dynamic orthodontic models are frequently required to ensure accuracy and better adjustment. Some research has shown the rigor, efficiency, consistency, and reproducibility of orthodontic models produced by additive manufacturing (AM) and 3D printers. Orthodontic models produced by AM and 3D printers are proven suitable for clinical use. Various methods, such as intraoral scanning, re-scanning existing plaster models, or comparing with a typodont model, are used to formulate 3D printed orthodontic models [27,28,29,30,31,32]. For example, in an experimental study, Anita Fekonja and colleagues tested and evaluated additively manufactured clear aligners for solving orthodontic malocclusions. They designed the crown using 3D dental design software and produced it using selective laser melting (SLM) technology. They used a custom-made fixed sagittal guidance (FSG) appliance that was fitted on the upper molars and compared it with the SLM-based molar and the traditionally constructed one. The results show the great potential and adaptability of AM in orthodontic treatment [33]. AM and 3D printing technologies can increase the accuracy and reproducibility of dental casts, especially in mechanical aspects such as the base design. AM-based techniques do not affect the accuracy of the layer height or the position of the 3D-printed template-generated model [34]. Additive manufacturing (AM) also supports the creation of personalized orthodontic appliances, such as in aligners and retainers: Digital workflows enable the production of clear aligners with a precise fit.

Also AM has improved the fabrication in orthodontic space maintainers where custom-fitting devices can be printed quickly, which is especially beneficial in pediatric cases [35].

### 4.3. Pediatric Dentistry

Additive manufacturing (AM), commonly referred to as 3D printing, is revolutionizing pediatric dentistry by enabling the production of highly customized devices and educational tools tailored to the unique anatomical, developmental, and psychological needs of young patients. The integration of digital workflows, including intraoral scanning, computer-aided design (CAD), and computer-aided manufacturing (CAM), enhances precision, efficiency, and patient experience in pediatric dental care [36,37]. AM technologies such as stereolithography (SLA), digital light processing (DLP), and fused deposition modeling (FDM) are particularly valuable due to their ability to produce high-resolution, biocompatible devices with minimal material waste [38,39].

Space maintainers are crucial for preventing malocclusion and ensuring proper dental arch development in children who have experienced premature tooth loss. Traditional fabrication methods, such as manual casting, are time-consuming and often result in suboptimal fit [40,41]. AM addresses these challenges by enabling the rapid production of patient-specific space maintainers using biocompatible polymers, such as polylactic acid (PLA) or photopolymer resins. Digital workflows, which combine intraoral scanners and CAD software (DentalCAD; exocad GmbH), enable clinicians to design maintainers that precisely conform to a child’s oral anatomy, thereby reducing discomfort and improving clinical outcomes [41,42]. A study by Tamburrino et al. demonstrated that 3D-printed space maintainers, fabricated using digital light processing (DLP) with a biocompatible photopolymer resin (“OD-Clear MF Bio monomer free”), achieved high dimensional accuracy and precise fit, significantly outperforming conventional band-and-loop space maintainers in terms of fit and fabrication speed [43]. The study employed a fully digital workflow that involved intraoral scanning, generative design, and DLP-based additive manufacturing, resulting in space maintainers with smooth surface finishes and accurate geometries that conformed to the patient’s dental anatomy without clearance or interference. The digital approach reduced fabrication time compared to traditional manual methods, as it eliminated the need for extensive band selection and manual adjustments, thereby minimizing chair time, a critical factor in pediatric dentistry where patient cooperation is paramount [43].

Pediatric patients frequently experience anxiety during dental visits, which can hinder treatment compliance. 3D-printed anatomical models of teeth, jaws, and oral structures serve as educational tools to demystify procedures, thereby reducing fear and enhancing patient engagement. These models, typically produced using cost-effective FDM with PLA or multi-material PolyJet for realistic textures, allow clinicians to visually explain treatments to children and their guardians in an age-appropriate manner [44,45]. A narrative review by Kasihani and Rikawarastuti found that 3D-printed models improved children’s understanding of procedures by 40%, leading to reduced anxiety and increased cooperation [44]. These models are also valuable in parental education, fostering informed consent and trust [44]. Additionally, 3D-printed models are utilized in preclinical training, allowing dental students to practice pediatric procedures on replicas of complex anatomies, thereby enhancing procedural confidence and competence [44,46].

Despite its advantages, AM in pediatric dentistry faces several challenges. Ensuring the biocompatibility of materials for prolonged intraoral use is critical, as residual monomers from incomplete resin curing can pose cytotoxic risks [21]. The high initial cost of 3D printers and digital imaging systems can be prohibitive for small or rural clinics, exacerbating access disparities [47]. Regulatory compliance for pediatric devices necessitates stringent testing to ensure safety, particularly for long-term applications such as space maintainers or restorations [22]. Additionally, the adoption of AM necessitates training in digital workflows, which may be lacking among pediatric dentists.

The future of AM in pediatric dentistry lies in integrating bioprinting and regenerative technologies. Hydrogel-based bioinks, embedded with dental pulp stem cells or growth factors, could enable the regeneration of pulp tissue in young permanent teeth, thereby revolutionizing pulpal therapy [48]. The convergence of 3D printing with virtual reality (VR) and augmented reality (AR) presents opportunities for immersive patient education and procedural simulations, thereby further reducing anxiety [49]. Longitudinal clinical trials are necessary to validate the durability and safety of 3D-printed devices in pediatric populations, alongside efforts to develop cost-effective and sustainable materials that enhance accessibility.

### 4.4. Endodontics

Additive manufacturing is transforming endodontics by enabling the production of precise, patient-specific tools, obturation systems, and regenerative scaffolds that address the complexities of root canal anatomy. The integration of 3D printing with cone-beam computed tomography (CBCT) enhances the accuracy and efficiency of endodontic procedures, improving clinical outcomes and reducing procedural errors [50]. Technologies such as SLA, DLP, and PolyJet are widely used for their high resolution and compatibility with biocompatible materials, facilitating applications ranging from guided endodontics to tissue engineering. This section examines the role of AM in endodontics, its current applications, challenges, and emerging trends.

An in vitro study by Huth et al. compared the accuracy of root canal localization in 3D-printed teeth using dynamic navigation, static guides, and freehand techniques [51]. Static guides showed the least angle deviation (1.12 ± 0.85°), followed by dynamic navigation (2.82 ± 1.8°), and freehand (9.53 ± 6.36°). Dynamic navigation minimized tooth substance loss, while freehand caused the most. The operating time was shortest for the freehand method, followed by the static and dynamic methods. Guided approaches enhance precision and conserve tooth structure [51]. Another in vitro study evaluated the accuracy and root surface temperature changes during guided endodontic access cavity preparation using 58 single-rooted premolars. CBCT and intra-oral scans guided the design of 3D-printed plates for 40 teeth, with a custom bur used for access. Postoperative CBCT showed minimal deviations: 0.30 mm (tip) and 0.28 mm (base) mesial/distal, 0.28 mm (tip) and 0.25 mm (base) buccal/lingual, with an angle deviation of 3.62°. Temperature changes in 18 teeth, divided into three groups (guided endodontics, ET20, ProTaper F3), showed guided endodontics had the lowest mean root surface temperature rise (5.07 °C, *p* < 0.05). Guided endodontics offers feasible accuracy and minimal temperature increase; however, in vivo studies are needed to confirm its clinical reliability [52].

Regenerative endodontics aims to restore pulp vitality in immature permanent teeth, and AM plays a pivotal role by producing scaffolds for tissue engineering. Hydrogel-based bioinks, such as gelatin methacryloyl or sodium alginate combined with cellulose nanocrystals, are used to print scaffolds that support the proliferation of dental pulp stem cells (DPSCs) and promote revascularization [48]. Results of a study demonstrated that 3D-printed microgels successfully regenerated pulp-like tissue in vitro, with potential applications in revitalizing necrotic teeth. These scaffolds can be embedded with growth factors or drugs for controlled release, enhancing therapeutic efficacy [53].

3D-printed endodontic models replicate complex root canal anatomies for preclinical training, offering realistic tactile feedback and radiopacity. Produced using PolyJet technology with multi-material resins, these models simulate the properties of dentin and pulp, enabling students to practice access preparation and canal instrumentation [44,54,55]. A study by Reymus et al. reported that dental students trained on 3D-printed endodontic models achieved a 22% higher proficiency in root canal preparation compared to those using extracted teeth, highlighting the educational value of these models in preclinical training [56].

Another study by Ho et al. demonstrated that artificial incisor and molar models, fabricated using a self-developed multi-resin 3D printer with radiopaque A2 resin (incorporating 10% barium sulfate) for the tooth body and soft red resin for the pulp cavity, closely replicated the appearance, size, shape, and radiopacity of natural human teeth [55]. These models, created from micro-computed tomography data, improved trainee confidence and procedural accuracy in root canal treatment, suggesting an increased clinical success rate compared to conventional training models. The use of PolyJet technology enabled the precise differentiation of enamel, dentin, and pulp, providing realistic tactile feedback and radiographic visualization that are critical for practical endodontic training [55].

Ensuring the biocompatibility of printed materials, particularly for regenerative applications, requires rigorous testing to address cytotoxicity risks from residual monomers [57]. Regulatory frameworks for custom endodontic devices vary by region, which complicates the approval process [22].

Future advancements in endodontics will likely focus on bioprinting for whole-pulp regeneration, with bioinks engineered to mimic the extracellular matrix of dental pulp [53]. AI-driven design optimization, coupled with finite element analysis (FEA), could enhance the precision of endodontic guides and obturators by predicting biomechanical performance. A study highlighted that AI-driven tools, integrated with FEA, enable precise design of 3D-printed endodontic guides, improving canal access accuracy and biomechanical stability, offering transformative potential for personalized endodontic treatments [58].

The integration of VR/AR with 3D-printed models may enable real-time procedural simulations, improving training and treatment planning [49]. Longitudinal clinical trials are crucial for validating the durability and safety of 3D-printed endodontic devices, thereby paving the way for broader adoption.

### 4.5. Implantology

Additive manufacturing has transformed dental implantology by enabling the production of patient-specific implants, surgical guides, and restorative frameworks with unparalleled precision and biocompatibility. Technologies such as selective laser sintering (SLS), SLA, and DLP, combined with advanced imaging (CBCT) and computational modeling, optimizing implant design and placement, improving osseointegration, aesthetics, and long-term success [59,60]. This section explores the applications of AM in implantology, its challenges, and future innovations driving personalized and regenerative dental care.

3D-printed surgical guides are a cornerstone of modern implantology, enhancing the accuracy of implant placement. Fabricated using SLA or DLP with biocompatible resins, these guides are designed from CBCT scans to ensure precise drill positioning, angulation, and depth, minimizing risks of nerve damage or sinus perforation [61]. A meta-analysis by Tahmaseb et al. reported that 3D-printed guides achieved a mean angular deviation of less than 2 degrees and a linear deviation of 0.5 mm, significantly outperforming freehand techniques. The incorporation of press-fit metal bushings enhances drill stability, ensuring precise alignment with planned implant sites [62]. These guides are particularly valuable in complex cases, such as full-arch rehabilitation or patients with limited bone volume.

AM enables the fabrication of bespoke implants and abutments tailored to individual anatomies, improving osseointegration and aesthetic outcomes. Titanium and cobalt-chromium alloys, printed using SLS, offer high strength, corrosion resistance, and biocompatibility for permanent implants [17]. A study by Iezzi et al. evaluated a 3D-printed Ti-6Al-4V dental implant with a porous, acid-etched surface (TEST) compared to a machined surface (CTRL) [63]. Scanning electron microscopy revealed a highly interconnected porous architecture and rough surface in the TEST group. The TEST implants showed enhanced adhesion and proliferation of human oral osteoblasts (hOBs), increased expression of osseointegration biomarkers, and slightly higher collagen production. Human adipose-derived mesenchymal stem cells (hAD-MSCs) exhibited greater expression of endothelial and osteogenic markers. At the same time, monocytes showed increased synthesis of anti-inflammatory M2 phenotype mRNA, indicating the potential of these 3D-printed implants for future clinical use [63].

Custom abutments, printed with zirconia or PEEK, ensure precise marginal fit and aesthetic integration with soft tissues [5]. It has also been reported that 3D-printed zirconia abutments exhibit color stability and marginal accuracy within 50 μm, matching those of milled restorations [64].

The use of lattice structures in 3D-printed implants, designed via finite element analysis (FEA), mimics the porosity of natural bone, reducing stress shielding and promoting bone ingrowth. SLS-printed titanium implants with lattice designs have demonstrated enhanced biomechanical stability in animal models, characterized by increased bone-implant contact ratios [65]. AM enables the design of tailored scaffolds with customized geometries, mechanical properties, and biological responses, thereby addressing the variability of bone defects. The study emphasizes the importance of interdisciplinary collaboration among patients, clinicians, and engineers, integrating bone defect imaging, material selection, topography design, and fabrication methods to optimize integration and wound healing, advancing personalized medicine beyond traditional implant solutions [59].

AM supports the production of provisional crowns, bridges, and frameworks for implant-supported restorations using polymers like PEEK or ceramics like zirconia. These restorations, printed using DLP or SLA technology, ensure precise occlusion and aesthetics during the healing phase [66]. A study by Rues et al. compared the fit of 3D-printed zirconia veneers to milled zirconia veneers on resin-replicated maxillary central incisors prepared for thin veneers [67]. Cement gap sizes were measured at the margin, labial surface, and incisal edge. Milled veneers showed a better marginal fit, but 3D-printed veneers provided a superior fit at the incisal edge, as they eliminated the need for drill compensation, offering greater geometrical flexibility and clinically acceptable adaptation [67].

FEA and AI-driven modeling play a critical role in optimizing implant design. Quantitative computed tomography-based FEA frameworks, validated with digital image correlation, enable patient-specific simulations of stress distribution and load transfer, reducing failure risks [68]. Machine learning models trained on FEA data can predict implant performance and optimize lattice structures, expediting the design process. These tools enhance the precision of implant geometry and material selection, ensuring compatibility with individual biomechanical needs [69,70].

The high cost of metal 3D printers and CBCT systems limits accessibility, particularly in resource-constrained regions [71]. Regulatory approval for custom implants is complex, as it requires compliance with FDA or CE mark standards, which vary by region. The long-term durability of lattice-structured implants lacks extensive clinical data, necessitating further research [72]. Additionally, the integration of AM requires clinicians to acquire expertise in digital workflows, which may face resistance due to training gaps.

The integration of intelligent sensors into 3D-printed implants, enabled by advances in printed electronics, could allow real-time monitoring of osseointegration and biomechanical performance [73]. The convergence of AM with VR/AR technologies will enhance surgical planning and training, enabling immersive simulations of implant procedures [74,75]. Longitudinal clinical trials and standardized regulatory frameworks are crucial for validating the safety and efficacy of these innovations, thereby ensuring their integration into routine practice.

### 4.6. Maxillofacial and Oral Surgery

In oral oncology, 3D printing enables the development of personalized surgical guides and prosthetics, enhancing surgical accuracy and patient-specific treatment planning [76]. Complex anatomical structures can be replicated for surgical planning and reconstruction. Anatomical Models are used for preoperative planning and patient education [77]. Biocompatible scaffolds support tissue regeneration in reconstructive surgeries [48].

3D printed dental surgical guides are a prime example of precision instruments used in clinical settings within the medical domain. The distinctive anatomy of everyone is meticulously analyzed and crafted, resulting in the development of precise clinical guides that significantly enhance the accuracy and effectiveness of reconstructive procedures. This modeling approach is particularly beneficial in dental operations, where teeth serve as dependable and uniform reference points to ensure accurate placements. Surgical guides created through 3D printing with biocompatible resins are utilized to precisely determine the sites and depths necessary for dental implant drilling. Such a technique ensures that alignment with the existing dentition is maintained for optimal positioning.

Furthermore, press-fit metal bushings are integrated into the 3D printed design to ensure the drill bit is directed with precision. Generally, 3D-printed surgical guides necessitate the inclusion of bone or teeth; however, alternative reference points may also prove viable. Depending on the specific situation, 3D modeling software can be utilized to create a suitable model that accurately meets clinical objectives [78].

### 4.7. Dental Education and Simulation Training

3D-printed models have proven to be valuable tools in dental education, particularly in preclinical training, where they enhance student comprehension, procedural confidence, and engagement with complex anatomical structures [44]. Three-dimensional (3D) printed anatomical models are increasingly used in medical education to simulate complex procedures, providing hands-on training opportunities that enhance clinical skills and confidence [79].

### 4.8. Supplies for the Dental Clinics and Their Architectural Layout

Dental clinics face a heightened risk of COVID-19 transmission and cross-infection because many of the tools used during procedures generate aerosols, along with droplets of saliva, secretions, and blood. These substances can facilitate the spread of the virus between dental professionals and their patients [80]. During previous pandemics, there were mass stockpiles of drugs, personal protective equipment (PPE), and vaccines because of panic and increased demand. This may lead to mass catastrophes for people in need of immediate PPEs, drugs, and vaccines [81,82]. We can find new PPEs, drugs, and vaccines in the future more quickly by utilizing 3D printing technologies to overcome sudden shortages and meet high demand [83,84]. Like the medical treatments, shortages of personal protective equipment (PPE) have disrupted dental treatments during the COVID-19 pandemic. Since the COVID-19 pandemic, 3D-printed nasopharyngeal swabs, valves, PPEs such as face shields and facemasks, and many more medical devices have provided sufficient supplies in both medical and dental healthcare facilities. Therefore, 3D printing technologies can be utilized to produce an unlimited supply of PPEs and supplies for future dental care [84,85].

## 5. Innovations and Emerging Trends

### 5.1. 4D Printing and Smart Materials

4D printing introduces the dimension of time to 3D-printed objects, enabling them to change shape or function in response to external stimuli such as temperature, moisture, or pH. This innovation is up-and-coming for dental and biomedical applications. When exposed to specific stimuli, shape-memory polymers (SMPs) can return to their predefined shape, making them ideal for use in orthodontic devices and tissue [8]. Hydrogels and other innovative smart biomaterials are being explored for applications in regenerative dentistry and drug delivery systems [86].

These materials offer the potential for dynamic dental appliances that adapt to the oral environment, enhancing comfort and functionality. Recent studies have further emphasized the evolution of medical 3D printing and its expanding role in prosthetic and regenerative dental applications, highlighting the integration of printable biomaterials tailored for clinical use [87]. Inkjet printing technologies, initially developed for pharmaceutical applications, are now being explored in dentistry for their potential to deliver precise, patient-specific drug dosages and bioactive coatings on dental devices [88]. Emerging research also explores the integration of 3D-printed electronics into biomedical devices, paving the way for innovative dental applications such as responsive drug delivery systems and diagnostic tools [89]. The precision and personalization enabled by 3D printing in drug delivery systems are now being explored in dentistry, particularly for intraoral applications that require controlled release and patient-specific dosing [90]. Recent advances in 3D printing for drug delivery are shaping the future of personalized healthcare, with applications extending into dentistry, including the development of custom therapeutic devices and on-demand intraoral drug delivery systems [91]. Innovations in 3D printing for drug delivery pave the way for personalized intraoral therapeutic systems in dentistry, enabling precise, on-demand treatment tailored to individual patient [91].

In the field of craniofacial studies and progressive biomedical innovations, room temperature ionic liquids (RTILs) are emerging as promising multifunctional material for 3D printing dental products and toothpaste, owing to their unique physical and chemical properties, antimicrobial characteristics, precise task specificity, and environmental sustainability. Utilizing cutting-edge 3D bioprinting methods, there is a vast potential for integrating different quantities of room-temperature ionic liquids (RTILs) into toothpaste and mouthwash formulations to enhance the protective environment of oral cavities. Hayashi and colleagues recently examined an innovative method that incorporates ionic liquids into the formulation of fluoride-containing toothpaste. Their research led to the creation of a pioneering type of hybrid ionic liquids known as “fluoride ion-encapsulated germoxane cages,” which house a fluoride ion internally [92,93].

### 5.2. Computational Modeling

While AM is transforming dental science by enabling the development of patient-specific implants, prosthetics, and restorations with unprecedented precision and complexity, a key tool in the evolution of this field is computational modeling, more specifically, finite element analysis (FEA). It allows researchers and clinicians to simulate and optimize the biomechanical performance of these custom devices before they are ever placed in a patient.

FEA has been pivotal in understanding how implant geometry, thread design, and material choice affect stress transfer to the surrounding bone [94]. FEA was utilized to demonstrate that the 3D-printed composites are more suitable for temporary applications, as they exhibit higher stress concentrations and lower fracture resistance [95].

Recent advancements in dental science are making personalized care more achievable than ever. Quantitative computed tomography-based finite element (QCTFE) modeling, when validated with digital image correlation (DIC) systems, allows researchers and clinicians to create highly accurate, patient-specific digital models [96]. This approach enables dental professionals to predict how an implant or restoration will behave in a particular patient’s mouth, leading to more precise and reliable treatments. An open-source platform and workflow have been developed that are both extensible and easily accessible to the research community. This system accounts for the natural variation in mechanical properties throughout the body, enabling the simulation and analysis of how various dental implant designs or materials may perform for individual patients [97]. With these tools, researchers can examine potential failure points and optimize the geometry and materials of dental implants, ultimately improving patient outcomes and advancing personalized dental care.

Furthermore, the biomechanical behavior of zirconia, lithium disilicate (IPS e.max CAD), and a 3D-printed composite (VarseoSmile CrownPlus; BEGO, Bremen, Germany) for maxillary anterior bridge restorations were compared using FEA, where the stress distribution and material behavior were examined in detail [95]. The use of lattice structures in additively manufactured implants has been explored to mimic the natural degradation of bone stiffness and meet patient-specific requirements, reducing stress shielding and promoting bone ingrowth [65,98,99].

While these computational tools facilitate the design of highly sophisticated and patient-specific geometries, their successful implementation as clinically effective implants is fundamentally contingent upon the intricacies of the additive manufacturing process itself. This presents a notable limitation: the optimal performance forecasted by finite element analysis (FEA) can be compromised if the printing parameters are not meticulously optimized. The mechanical properties and structural integrity of the final product are directly influenced by a complex array of process variables, including material viscosity, build orientation, and energy source settings, which are often determined through empirical and labor-intensive methods.

In the fabrication of the 3D-printed composite bridges referenced, the post-curing strategy constitutes a crucial parameter [100]. Variations in ultraviolet (UV) light exposure duration and intensity can significantly influence the extent of polymer cross-linking, thereby affecting the material’s hardness, fracture toughness, and wear resistance. The relationship between manufacturing parameters, such as post-curing strategies, and Finite Element Analysis (FEA) is direct and crucial for achieving precise simulation outcomes. The predictive reliability of an FEA model hinges on the accurate assignment of material properties within its constitutive framework. These properties—such as Young’s modulus, fracture toughness, and yield strength—are not fixed constants but are influenced by the fabrication and post-processing procedures employed.

Consequently, the post-curing process, which governs the degree of polymer cross-linking, ultimately determines the final mechanical properties of the composite restoration. For FEA simulations to yield clinically relevant insights, material data must be empirically derived from samples produced under identical conditions as the final component. Any discrepancy between the material properties assumed in the digital model and those of the actual part could undermine the validity of stress and strain predictions, thus compromising the analysis’s reliability. Therefore, meticulous control of fabrication parameters is essential to ensure the validity and accuracy of biomechanical simulations.

### 5.3. AI and Digital Workflow Integration

Artificial intelligence (AI) is increasingly integrated into the digital workflow of dental practices, streamlining processes and improving precision. Machine learning algorithms optimize dental prosthesis design, predict printability, and enhance treatment planning [101]. Digital impressions captured via intraoral scanners are processed using CAD software, enabling rapid and accurate restorations [15]. Simulation-based analyses have demonstrated that discrete-event modeling can optimize additive manufacturing workflows in dental clinics, thereby improving resource utilization and patient throughput [102]. AI supports automation in slicing, error detection, and print parameter optimization, reducing human error and improving efficiency [103].

Additionally, FEA can be used to build a virtual laboratory, generating high-fidelity data that trains machine learning (ML) models to optimize implant material properties and geometry. This approach enables rapid, data-driven design improvements, reducing the need for extensive physical testing. Recent studies have demonstrated that machine learning (ML) models trained on finite element analysis (FEA) data can accurately predict implant performance, optimize lattice structures, and expedite the design process [104,105]. These technologies transform dental practices into highly digitized environments, resulting in improved patient outcomes and reduced chair time.

Furthermore, the scope of this digital optimization transcends implant geometry and extends to the manufacturing process itself. The successful fabrication of dental implants through additive manufacturing is critically reliant on a complex interaction of printing parameters. Determining the optimal combination of these variables presents a substantial challenge, as they are frequently interdependent and directly influence the microstructure, mechanical integrity, and surface finish of the final component. This constraint implies that even a meticulously designed implant may fail if the printing process is not meticulously controlled and customized to the specific material and application.

For example, in vat photopolymerization methods, parameters such as light intensity, layer exposure duration, and post-curing procedures are of critical importance [106]. Modifying the curing process can drastically alter the degree of polymerization, which in turn dictates the material’s final strength, hardness, and biocompatibility. Similarly, for powder bed fusion methods, variables such as laser power, scan speed, and layer thickness need to be carefully adjusted to reduce porosity and residual stresses stress [107]. The same machine learning models trained on FEA data can therefore be applied not only to optimize the design, but also to predict how these varying printing parameters will affect the final outcome, thus overcoming the limitations of traditional, empirical trial-and-error methods.

### 5.4. Leadership Strategies for Productivity with 3D Printing

Recent qualitative research by Zamanian highlights how dental center leaders in the United States have successfully leveraged on-site 3D printing to enhance productivity and patient care [108]. Grounded in the theory of disruptive innovation, the study identified three core strategies:

1. Enabling technology strategy, which focuses on integrating digital workflows and training staff.
2. Innovative business model strategy, which includes in-house production to reduce outsourcing and turnaround times.
3. The customer demand strategy focuses on enhancing the patient’s experience by reducing chair time and minimizing the number of visits.

These strategies streamline operations and contribute to broader social outcomes, such as expanding access to affordable dental care and creating local employment opportunities.

### 5.5. Additive Manufacturing and 3D Printing in Dental Practice as a Driver of Environmental Sustainability and Circular Economy

Environmental sustainability is becoming a priority in dental manufacturing, with 3D printing offering several eco-friendly advantages:

- Recycled materials and eco-friendly practices: Studies have shown that recycled nylon and other polymers can be effectively reused in dental applications without compromising quality [11]. Additive manufacturing reduces material waste compared to subtractive methods and supports on-demand production, minimizing inventory and transportation emissions [109].
- Circular economy models and cost reduction: Hospitals and clinics are exploring closed-loop systems that convert waste plastics into usable filaments for printing anatomical models and devices. 3D printing also promises to reduce healthcare costs by enabling on-demand production, minimizing material waste, and streamlining supply chains, contributing to more sustainable and cost-effective dental care [110].
- Use of sustainable and biocompatible materials: New dental 3D printing materials are being developed, including biocompatible, recyclable, and bio-based polymers like polylactic acid (PLA) composites made from renewable biomass. Compared to petroleum-derived resins, these options are more environmentally friendly and align with circular economy principles when used in closed-loop recycling systems [111,112,113].
- Energy efficiency and process optimization: Although additive manufacturing (AM) consumes electricity during printing and post-curing, innovations such as stereolithography apparatus (SLA) allow the creation of complex structures with high precision. SLA also offers benefits like high efficiency and energy savings, helping to cut energy use without sacrificing quality [114,115]. Original research by Caelli and colleagues showed that using direct printing through Additive Manufacturing (AM) offers environmental benefits, mainly due to decreased raw material use and lower electricity consumption [116]. When combined with renewable energy sources, these technologies can further reduce the carbon footprint of dental manufacturing.
- Environmental assessment for continuous improvement in dental practice: Using life cycle analysis (LCA) methods, dental practices can measure the environmental impact of various manufacturing approaches. This evidence-based strategy supports ongoing workflow improvements aimed at achieving sustainability goals while upholding high clinical quality. A 2024 study using the LCA method found that dental practices have increased their overall carbon footprint (CFP) from 27 to 35 tons, primarily due to higher staff travel and waste generation. The incineration of mixed dental waste contributes around fifteen hundred kg of carbon emissions per ton, underscoring the significant environmental impact of waste management and the need for more sustainable disposal methods. They recommended addressing waste and promoting low-carbon transport within the dental practice, which is vital [117]. According to a recent study from Egypt, private dental laboratories contribute significantly to carbon emissions, mainly from staff travel. This results from the reliance on several couriers in each laboratory to deliver impressions, prostheses, and appliances [118]. With on-site 3D printing and AM, dental clinics can manufacture crowns, aligners, and occlusal splints internally, lessening reliance on centralized labs for delivery. This shift toward localized production helps lower packaging waste and transport-related emissions, promoting greener dental supply chains. Utilizing life cycle analysis (LCA) methods should be routinely checked for dental practices to monitor their carbon footprint emissions.

User experience studies have highlighted that while 3D printing enhances workflow efficiency and customization in dentistry, its long-term sustainability depends on factors such as material reuse, energy consumption, and practitioner training [119].

## 6. Challenges and Limitations

Despite the transformative potential of 3D printing in dentistry, several challenges and limitations hinder its widespread adoption and optimal use:

*Material Limitations*

- Biocompatibility and Mechanical Properties: Not all printable materials meet the stringent requirements for long-term use in the oral cavity. Some resins may release residual monomers, posing cytotoxic risks [21].
- Limited Material Diversity: Although polymers, metals, and ceramics are utilized, the range of materials suitable for specific dental applications remains narrow, particularly for permanent restorations [8].
- Post-Processing Needs: Many materials require extensive post-curing, cleaning, and finishing to achieve desired properties, increasing complexity and time [15]. Surface optimization in fused filament fabrication has been shown to significantly impact the quality of dental implants, with parameters like layer thickness and build orientation playing a crucial role in achieving smoother finishes and better clinical outcomes [120].

*Cost and Accessibility*

- High Initial Investment: Equipment, software, and materials can be expensive, especially for small or rural clinics [3].
- Maintenance and Upgrades: Regular calibration, updates, and repairs add to operational costs.
- Economic Disparities: Access to advanced 3D printing technologies is uneven across regions and institutions, limiting equitable adoption [47].

*Regulatory and Standardization Issues*

- Lack of Unified Standards: Variability in material quality, printer performance, and post-processing protocols complicates regulatory approval [86].
- Regulatory Complexity: Navigating FDA or CE mark requirements for custom medical devices can be time-consuming and costly [22].
- Quality Assurance: Ensuring consistent output across different printers and batches remains a significant challenge.

*Training and Adoption Barriers*

- Skill Gaps: Effective use of 3D printing requires knowledge of CAD/CAM, material science, and digital workflows, which many practitioners lack [121].
- Resistance to Change: Some clinicians hesitate to adopt new technologies due to perceived complexity or disruption to established workflows.
- Educational Integration: The limited inclusion of 3D printing in dental curricula hinders the development of a digitally fluent workforce [122].

## 7. Future Directions

### 7.1. Personalized and Regenerative Dentistry

The future of dental care is moving toward highly personalized treatments and biologically integrated solutions. Patient-specific restorations and prosthetics are becoming increasingly precise with the use of digital workflows and 3D printing [15]. Regenerative dentistry utilizes bioactive materials and scaffolds to stimulate tissue healing and regeneration, particularly in the fields of periodontics and endodontics [48]. Emerging applications of 3D printing in medicine, such as tissue engineering and patient-specific surgical planning, are increasingly influencing dental innovations, particularly in regenerative and personalized care [123]. 3D printing technologies increasingly enable the development of customized implants and controllable biosystems, offering new possibilities for precision dental therapies and regenerative applications [124].

### 7.2. Bioprinting of Dental Tissues

Bioprinting is emerging as a transformative technology in dental tissue engineering. Hydrogel-based bioinks are being developed to print scaffolds that support the growth of dental pulp stem cells [48]. Advancements in 3D printing technologies continue to drive innovation in medical materials, particularly in the development of biocompatible scaffolds and drug delivery systems, which hold promising implications for regenerative and restorative dental applications [125]. Innovations in 3D biomaterial printing enable the fabrication of functional, tissue-compatible constructs, laying the groundwork for future applications in regenerative dental therapies [126]. Future applications include bio-printed periodontal ligaments, dentin-pulp complexes, and even whole teeth, providing comprehensive solutions for complex oral tissue loss.

### 7.3. Integration with Virtual and Augmented Reality

The convergence of 3D printing with VR/AR technologies enhances clinical and educational experiences. Virtual surgical planning and augmented reality overlays enable clinicians to simulate procedures and visualize anatomical structures in real-time [49]. Dental education benefits from immersive simulations that utilize 3D-printed models combined with augmented reality (AR) for hands-on learning [44]. 3D printing accelerates healthcare innovation by reducing prototyping costs and enabling rapid development of patient-specific solutions, a trend increasingly reflected in dental research and practice [127].

### 7.4. Clinical Validation and Long-Term Studies

To ensure safe and effective integration into routine practice, future efforts must focus on Longitudinal clinical trials and standardization, as well as the development of regulatory frameworks. Longitudinal clinical trials to assess the durability, biocompatibility, and patient outcomes of 3D printed restorations and implants [128]. Standardization and regulatory frameworks to support widespread adoption and ensure consistent quality across devices and materials [86].

## 8. Conclusions

Additive manufacturing (AM) has become a revolutionary element in dentistry research, connecting digital innovation with clinical applications. Technologies such as SLA, FDM, SLS, DLP, and PolyJet enable the precise production of customized dental restorations, surgical guides, prosthetics, and teaching models, thereby enhancing efficiency and reducing turnaround times. The selection of materials, ranging from biocompatible polymers such as PLA, PMMA, and PEEK to high-strength metals like titanium and cobalt-chrome, and aesthetically superior ceramics like zirconia and glass ceramics, has dramatically broadened the applications of additive manufacturing in dentistry. These developments facilitate various applications in prosthodontics, orthodontics, implantology, maxillofacial surgery, and pediatric care. Notwithstanding its potential, specific issues must be addressed to promote wider implementation. This encompasses ensuring material biocompatibility, navigating regulatory difficulties, addressing substantial initial expenses, and bridging the digital skills gap among practitioners. Moreover, environmental sustainability is becoming increasingly significant, with a focus on the utilization of recycled resources, waste reduction, and circular economy techniques. Forthcoming technologies, including 4D printing, AI-based design optimization, virtual reality integration, and bioprinting of dental tissues, are set to transform dental treatment by improving personalization, functionality, and patient outcomes. These breakthroughs will necessitate interdisciplinary collaboration, clinical validation, and educational reform to incorporate AM seamlessly into dental practice. Additive manufacturing represents a transformative revolution in dentistry, enabling accurate, individualized, and restorative treatments. Its ongoing development is poised to transform oral health delivery, diminish healthcare disparities, and stimulate innovation throughout all areas of dentistry science. As additive manufacturing continues to evolve, it holds the promise not only to enhance clinical precision but also to democratize access to high-quality dental care worldwide.

## Figures and Tables

**Table 1:** Comparative Analysis of Techniques.

Technology	Accuracy & Resolution	Speed	Cost	Best Suited for
SLA	Very high	Moderate	Moderate	Surgical guides, crowns, bridges [5]
FDM	Moderate	High	Low	Educational models, prototypes [15]
SLS	High	High	High	Durable prosthetics frameworks [12]
DLP	Very high	High	Moderate	Detailed restorations aligners [3]
PolyJet	High	Moderate	High	Multi-material models and training tools [13]

**Table 2:** Summary of primary category of materials used in 3D printing dental application.

Material Type	Biocompatibility	Strength	Aesthetics	Cost	Typical Applications
Polymers	High (e.g., PLA, PMMA, PEEK)	Moderate to High	Good (e.g., resins)	Low to Moderate	Crowns, Bridges, Surgical Guides
Metals	High (e.g., Titanium, Cobalt-Chrome)	Very High	Moderate	High	Implants, Frameworks
Ceramics/Composites	High (e.g., Zirconia, Alumina, Glass Ceramics)	High	Excellent	Moderate to High	Crowns, Bridges, Implant Abutments

## Data Availability

No new data were created or analyzed in this study.

## Abbreviations

The following abbreviations are used in this manuscript:

AM	Additive Manufacturing
3D	Three-Dimensional
4D	Four-Dimensional
UV	Ultraviolet
SLA	Stereolithography
FDM	Fused Deposition Modeling
SLS	Selective Laser Sintering
DLP	Digital Light Processing
CAD	Computer-Aided Design
CAM	Computer-Aided Manufacturing
PEEK	Polyether Ether Ketone
PLA	Polylactic Acid
PMMA	Polymethyl Methacrylate
AI	Artificial Intelligence
VR	Virtual Reality
AR	Augmented Reality
CBCT	Cone Beam Computed Tomography
FEA	Finite Element Analysis
ML	Machine Learning
RTIL	Room Temperature Ionic Liquid
PPE	Personal Protective Equipment
hOBs	Human Oral Osteoblasts
OD	Optical Density
SMPs	Shape-memory Polymers
pH	Potential of Hydrogen
PGFA	Gutta-percha-filled area
